# *Amanita muscaria* extract potentiates production of proinflammatory cytokines by dsRNA-activated human microglia

**DOI:** 10.3389/fphar.2023.1102465

**Published:** 2023-04-12

**Authors:** Ashley Wagner, Marcus Pehar, Zhimin Yan, Marianna Kulka

**Affiliations:** ^1^ Nanotechnology Research Centre, National Research Council Canada, Edmonton, AB, Canada; ^2^ Neuroscience and Mental Health Institute, University of Alberta, Edmonton, AB, Canada

**Keywords:** microglia, inflammation, toll-like receptor, trehalose, HMC3, HMC3 cells, cytokines

## Abstract

Recent interest in mushrooms and their components as potential therapies for mental health, along with recent government and health authority approvals, has necessitated a more comprehensive understanding of their effects on the cellular microenvironment of the brain. *Amanita muscaria* has been ingested as a treatment for a variety of ailments for centuries, most notably those affecting the central nervous system and conditions associated with neuroinflammation. However, the effects of these extracts on neuroinflammatory cells, such as microglia, are unknown. The effect of commercially-sourced *A. muscaria* extract (AME-1) on human microglial cell line (HMC3) expression of surface receptors such as CD86, CXCR4, CD45, CD125 and TLR4 was determined by flow cytometry. AME-1 upregulated expression of all of these receptors. The effect of AME-1 on HMC3 production of IL-8 and IL-6 was determined and compared to tumor necrosis factor (TNF), polyinosinic-polycytidylic acid [poly(I:C)], substance P and lipopolysaccharide (LPS), all known activators of HMC-3 and primary microglia. HMC3 produced both IL-8 and IL-6 when activated with LPS, TNF and poly(I:C) but not when they were activated with substance P. Although AME-1 at higher concentrations increased IL-8 production of HMC3 on its own, AME-1 notably potentiated HMC3 production of IL-8 in response to poly(I:C). AME-1 altered expression of toll-like receptor 3 (TLR3) mRNA but not surface protein by HMC3. AME-1 also did not significantly alter expression of retinoic acid-inducible gene I (RIG-I) or melanoma differentiation-associated protein 5 (MDA5), both cytosolic sensors of dsRNA. Metabolomics analysis showed that AME-1 contained several metabolites, including the autophagy inducer, trehalose. Like AME-1, trehalose also potentiated HMC3 poly(I:C) mediated production of IL-8. This study suggests that *A. muscaria* extracts can modify HMC3 inflammatory responses, possibly due to their trehalose content.

## Introduction

Naturally-derived substances have been proposed as effective therapies for sleep disorders and anxiety (Sienkiewicz-Jarosz et al., 2003; Zhang et al., 2014) and include compounds obtained from certain species of mushroom such as *A. muscaria* (fly agaric). These mushrooms are known to contain muscarine, muscimol, muscazone and ibotenic acid (Michelot and Melendez-Howell, 2003; Lee et al., 2018) and have been used as an inebriant in eastern Siberia for centuries. The chemical composition of these mushroom extracts is poorly characterised but likely contains many other compounds that could have effects on brain cells. The effect of *A. muscaria* on brain tissue and cells is unknown.

The brain is composed of different cell types including microglia which facilitate and modulate neuroinflammation. Microglia are resident immune cells of the central nervous system that regulate inflammatory responses in the brain (Lue et al., 2010). In *in vivo* models, primary microglia are typically stimulated by mediators such as tumor necrosis factor (TNF), lipopolysaccharide (LPS), and neuropeptides such as substance P (Ahn et al., 2022). Each of these mediators have slightly different effects on microglial function, but some of these mediators appear to at least change microglial receptor expression such as CD45, CD86, chemokine receptors, and toll-like receptors (TLR) (Townsend et al., 2004; He et al., 2021). Human microglia express the full-length isoform of the neurokinin receptor (NK-1R) that is activated by the neuropeptide, substance P, and human and mouse primary microglia functions are modified by substance P treatment in some models (Burmeister et al., 2017). The effect of substance P on microglia is complex, but may be associated with pain (Zieglgänsberger, 2019), neuroinflammation (Wang et al., 2017), opioid withdrawal (Tumati et al., 2012) and psychiatric disorders (Trépanier et al., 2016). Interestingly, substance P may mobilize a specific subpopulation of microglia in ischemic brain injury and protect the blood brain barrier (BBB), promoting recovery (Ahn et al., 2022). Therefore, unlike TNF and LPS, substance P seems to have an anti-inflammatory effect on responses involving primary microglia (Kim and Son, 2021).

HMC3 (also called CHME3, CHME-3, CHME-5, or C13-NJ) are a human microglial cell line with some characteristics resembling human brain microglia: they display a globular morphology with thick processes, appear to migrate to chemotactic factors and they can express CD11b and CD68 under certain conditions (Li et al., 2009), as well as the microglial specific markers P2RY12 and TMEM119^1718^. However, HMC3 do not express the astrocyte-specific marker, glial fibrillary acidic protein (GFAP) or the neuronal neurofilament marker NF70KD and, unlike primary cells, HMC3 are less responsive to LPS and do not express CD14 (Janabi et al., 1995). When stimulated with LPS, HMC3 have been shown to produce IL-6, IL-8, IL-12 and IFN-γ (Wang et al., 2021a). TNF activates upregulation of fibrosis-associated genes such as collagen and fibronectin by HMC3 (Wang et al., 2021b), and increases their expression of Iba-1, CD40 and CD14 (Wang et al., 2021a). The effect of substance P on HMC3 has not been characterized.

HMC3 are activated by viral infection and produce many mediators, including IL-8, in response to hepatitis C virus infection (Rajalakshmy et al., 2015). Interestingly, HMC3 are highly susceptible to rhinovirus infection such that human rhinovirus completes its entire viral cycle in HMC3 (Angel-Ambrocio et al., 2020). This presents the interesting hypothesis that perhaps HMC3 respond to viral analogs such as double-stranded RNA (dsRNA), which bind to and activate TLR3. Expression of the viral innate immune receptors toll-like receptors 3 (TLR3), melanoma differentiation-associated antigen 5 (MDA5) and retinoic acid-inducible gene I (RIG-I) by HMC3 have not been described.

Although HMC3 are an imprecise model of *in vivo* human microglia, they are an accessible model with which to understand the effects of various compounds on possible neuroinflammatory responses. The effect of *A. muscaria* extract on HMC3 is unknown. In particular, the effect of *A. muscaria* extract on stimulated HMC3 has not been examined. Although HMC3 can be infected with viruses, their responses to the TLR3 agonist polyinosinic:polycytidylic acid or poly(I:C), have not been reported or compared to other more common microglial activators such as LPS. The effect of poly(I:C) on primary microglia is complex. LPS and poly(I:C) stimulate production of TNF, IL-1β and IL-6 by primary mouse microglia (Pahan et al., 2023) and IL-10 in rat primary microglia (Scott et al., 2022). However, when poly(I:C) was injected intraperitoneally into the APP/PS1 mouse model of Alzheimer’s disease (AD), activation of TLR3 attenuated neuronal loss and inhibited the activation of microglia and astrocytes, decreasing the expression of proinflammatory mediators in the hippocampus (Wang et al., 2023a). Yet, poly(I:C)-activated animals are thought to mimic the pathological features of myalgic encephalomyelitis/chronic fatigue syndrome (ME/CFS) because they induce neuroinflammation and activation of microglia (Tamura et al., 2022). Therefore, we hypothesized that poly(I:C) may similarly activate HMC3 and activate the production of proinflammatory cytokines and that *A. muscaria* extract may modify these responses. We determined the effect of poly(I:C) on HMC3 IL-6 and IL-8 production and compared those responses to other known activators of microglia: TNF and LPS. We also compared HMC3 responses to substance P since substance P appears to activate microglia differently from TNF and LPS. Finally, we determined the effect of *A. muscaria* extract on HMC3 responses to LPS and poly(I:C), choosing one well-characterized and one less-characterized activator of HMC3 cells.

We show, for the first time, that HMC3 are activated by TNF and poly(I:C), but not substance P, to produce IL-6 and IL-8. We also show that while a commercially sourced *A. muscaria* extract (AME-1) had no effect on IL-6 production, it potentiated poly(I:C)-induced production of IL-8. This potentiating effect was not significantly associated with changes in the expression of TLR3, interferon regulatory factor 3 (IRF3), MDA5 or RIG-I, all associated with intracellular detection of dsRNA. Lastly, we show that trehalose, an autophagy inducer, and a major component of *A. muscaria* extract, also significantly potentiated IL-8 production by poly(I:C) activated HMC3. These data suggest that AME-1 activated HMC3 to produce significant amounts of cytokines and altered their surface receptor expression *via* signaling pathways that are likely different from other activators such as LPS.

## Materials and methods

### Materials

*Amanita muscaria* extract (AME-1) was provided by Psyched Wellness Inc. (Toronto, Ontario) and can be purchased directly from the manufacturer. AME-1 is a commercial product sold for human consumption.

### Human microglia cell (HMC3) culture

HMC3 cells were purchased from ATCC (Manasass, VA, United States). The cells were cultured in minimum essential medium (MEM) supplemented with 10% FBS (Hyclone, Logan, UT, US), 100 U/mL penicillin and 100 µg/mL streptomycin (Gibco, Waltham, MA, United States) and maintained in a humidified atmosphere of 5% CO_2_ in air at 37°C. The cells were trypsinized in 0.25% trypsin-EDTA (Gibco) and maintained at 2 × 10^4^ cells/cm^2^ once they reached maximum confluency.

### Treatments

Stock concentrations of LPS (1 mg/mL, Sigma, St. Louis, MO, US), poly(I:C) (10 mg/mL, Sigma), recombinant human TNF (100 µg/mL, ThermoFisher, Waltham, MA, United States), Trehalose (250 mM, Sigma) and AME-1 (diluted 1:2) were prepared in sterile phosphate buffered saline (PBS, Gibco) pH 7.4 without calcium or magnesium. Substance P (Bachem, Bubendorf, CH) was prepared in 0.1 M acetic acid to a concentration of 1 mg/mL. Working stocks of treatments were diluted in culture medium prior to addition to cells.

### Cytokine enzyme-linked immunosorbent assays (ELISAs) and electrochemiluminescent multiplex analysis

HMC3 were seeded at 0.4 × 10^6^ cells per well in a 6-well plate and incubated in a humidified atmosphere of 5% CO_2_ in air at 37°C overnight to adhere. For single treatments the cells were then treated with AME-1 (50–5,000 µg/mL), 1 µg/mL LPS (Sigma), 0.5 µg/mL poly(I:C) (Sigma), 0.25 µg/mL recombinant human TNF (ThermoFisher), 0.5 µg/mL substance P (Bachem) or 0.15–15 mM trehalose (Sigma) for 24 h at 37°C, 5% CO_2_ and cell free supernatants were collected. For co-treatments, the cells were treated with AME-1 (50–5,000 µg/mL), or trehalose (0.15–15 mM) for 3 h at 37°C, 5% CO_2_. Following incubation, the media was aspirated, replenished with fresh media and the cells were stimulated with poly(I:C) (0.5 µg/mL; Sigma), LPS (1 µg/mL; Sigma), substance P (0.5 µg/mL; Bachem), recombinant human TNF (0.25 µg/mL; ThermoFisher) or untreated for 24 h. Cell free supernatants were isolated. For ELISA analysis for human IL-8 and IL-6 commercial ELISA kits (R&D Systems, Minneapolis, MIN, US) were used and plates were read using a VarioSkan Lux plate reader (ThermoFisher). Electrochemiluminescent multiplex analysis was performed using a custom-made multiplex plate with capture antibodies specific for IL-1β, IL-4, IL-6, IL-8, IL-10, IL-12p70, IL-22, IFN-γ, CCL2 and TNF (Meso Scale Diagnostics LLC., Rockville, MD, United States) and the plate was read using a MESO QuickPlex SQ 120MM plate reader (Meso Scale Diagnositcs LLC.).

### Proliferation assays

HMC3 were seeded at 0.1 × 10^5^ cells/mL in a 96-well plate and incubated in a humidified atmosphere of 5% CO_2_ in air at 37°C overnight to adhere. The cells were then treated with AME-1 (50–5,000 µg/mL), poly(I:C) (0.5 µg/mL) (Sigma), LPS (1 µg/mL; Sigma), substance P (0.5 µg/mL) (Bachem), recombinant human TNF (0.25 µg/mL; ThermoFisher), trehalose (0.15–15 mM (Sigma), or untreated for 24 h. Metabolic activity was analysed using the Cell Proliferation Kit II (XTT Assay, Roche, Basel, Switzerland) according to the manufacturer’s instructions and the results are presented as percent metabolic activity relative to untreated cells.

### Trypan blue exclusion assays

HMC3 were seeded at 0.4 × 10^6^ cells/mL in a 6-well plate and incubated in a humidified atmosphere of 5% CO_2_ in air at 37°C overnight to adhere. The cells were then treated with AME-1 (50–5,000 µg/mL) for 24 h at 37°C in 5% CO2. Following incubation, the cells were trypsinized and mixed 1:1 with 0.4% Typan Blue (Gibco) and counted on a haemocytometer. Percent viable cells was calculated relative to untreated.

### Flow cytometric analysis of receptor expression

HMC3 were seeded at a density of 0.5 × 10^6^ cells/mL in a 6-well dish and incubated in a humidified atmosphere of 5% CO_2_ in air at 37°C for 2 h to adhere. Cells were treated with AME-1 (50–5,000 μg/mL), LPS (1 mg/mL), poly(I:C) (10 μg/mL), or trehalose (0.15–15 μM) for 24 h. The cells were removed with 0.25% trypsin-EDTA (Gibco), washed with PBS, blocked for 10 min with 3% bovine serum albumin (BSA) in PBS and subsequently labelled with mouse anti-human CD125-phycoerytherin (PE) (555902, BD Biosciences), mouse anti-human, CXCR4-PE (12–9999, Invitrogen), rat anti-mouse CD86-allophycocyanin (APC) (558703, BD Biosciences, Franklin Lakes, NJ, United States), mouse anti-human TLR4-FITC (sc-13593, Santa Cruz Biotechnology Inc., Dallax, TX, United States), or mouse anti-human CD45-fluorescein isothiocyanate (FITC) (A85816, antibodies-online Inc., Limerick, PA, United States) in 0.1% BSA in PBS for 1 h at 4°C. Cells were labelled with isotypes (IgG1-PE (568275, BD Biosciences), IgG2a-PE (12–4724–82, Invitrogen), IgG2a-APC (553932, BD Biosciences), or IgG2a-FITC (sc-2856, Santa Cruz Biotechnology Inc.) for comparison. Flow cytometry was performed using the CytoFLEX Flow Cytometer (Beckman Coulter, Brea, CA, United States) and data analysis was performed using FlowJo software version 10.6.1 (FlowJo LLC, Ashland OR, United States).

### qPCR analysis of TLR3 expression

HMC3 were seeded at a density of 1 × 10^6^ cells/mL and incubated in a humidified atmosphere of 5% CO_2_ in air at 37°C overnight to adhere. The cells were then treated with AME-1(50–5,000 µg/mL) for 3 h at 37°C in 5% CO2 and RNA was isolated using Qiagen RNeasy mini kit (Qiagen, Hilden, Germany). Complementary DNA was synthesized using 1 µg of total RNA and M-MLV reverse transcriptase (ThermoFisher). qPCR was performed using PrimeTime qPCR primer probes (IDT, Brighton, United Kingdom) for TLR3 and a StepOnePlus qPCR instrument (Applied Biosystems) Results are presented as ΔΔCT relative to untreated expression levels.

### Western blot analysis of TLR3, IRF3, MDA5 and RIG-I expression

HMC3 were seeded at a density of 0.5 × 10^6^ cells/mL in a 6-well dish and incubated in a humidified atmosphere of 5% CO_2_ in air at 37°C overnight to adhere. The cells were then treated with LPS (1 µg/mL, Sigma), poly(I:C) (0.5 µg/mL, Sigma), trehalose (0.15–15 μM, Sigma) or AME-1 (50–5,000 ug/mL) for 24 h. For cells treated with trehalose and poly(I:C), the cells were treated with trehalose (0.15–15 μM) for 3 h, the media was then removed and replaced with media containing 0.5 ug/mL poly(I:C) and the cells were incubated a further 24 h. The cells were then lysed in Laemmli buffer (50 mM Tris pH 6.8, 1% SDS, 9% glycerol, 1.8% β-mercaptoethanol, and 0.002% bromophenol blue) and boiled at 100°C for 15 min. Cell lysates were loaded on a 10% polyacrylamide gel and transferred to a nitrocellulose membrane. The membrane was subsequently blocked for 1 h at room temperature with 5% skim milk in TBS-T followed by incubation for1hr with 0.4 µg/mL of mouse anti-human TLR3 (sc-32232, Santa Cruz Biotechnology, Dallas, TX, United States), 0.5 µg/mL of mouse anti-human IRF3 (550428, BD biosciences), 2.5 µg/mL of rabbit anti-human MDA5 (33H12L34, Invitrogen), 1 µg/mL rabbit anti-human RIG-1 (PA510297, Invitrogen), or 1 µg/mL of mouse anti-β-actin loading control (PA1-183, Invitrogen) in 3% BSA-TBS-T, and finally incubated for 1 h with goat anti-mouse IRDye 680 (Li-Cor, Lincoln, NE, United States) or goat anti-rabbit IRDye 800 (Li-Cor) diluted 1:15,000 in 3% BSA-TBS-T. The membrane was imaged using a Li-Cor Odyssey CLx (Li-Cor). Densitometry data was generated using Image Studio software version 5.2 (Li-Cor) and presented as a ratio of the indicated band signal of treated samples to untreated samples and all samples are corrected to the signal of their respective β-actin band.

### Metabolomic profiling using NMR

AME-1 was diluted 100 times with D_2_O containing 0.15 mM TMSP (3-(trimethylsilyl)propionate-2,2,3,3-d4, Cambridge Isotope Laboratories, Inc., Tewksbury, MA, United States) for NMR measurement. NMR experiments were performed on a Varian Direct Drive VNMRS 600 spectrometer, operating at a magnetic field strength of 14.1 T (599.49 MHz proton frequency) and equipped with an autoX dual broadband probe. One dimensional (1D) ^1^H NMR spectra were measured for all samples using 1D ^1^H with water suppression sequence (NOESY 1D) at 298 K. TMSP was used as an internal reference standard for spectra calibration. Metabolites were identified and quantified using Chenomx NMR Suite 8.6 Professional (ChenomX Inc., Edmonton, Canada).

### Chromatographic conditions for HPLC-MS analysis

LC separation was performed using an Agilent Zorbax NH_2_ column (4.6 × 250 mm, 5 um) on an Agilent 1260 HPLC system. The mobile phase was composed of 20: 80 water: acetonitrile (EMD Millipore Corporation) with 2 mM ammonium acetate (HPLC grade, EM Science) at a flow rate of 1 mL/min. The volume of injection was 10 µL. Analyte detection was achieved using an Agilent Single-Quad MSD (G6135B) with an electrospray ionization (ESI) source. Identification and quantification were achieved by comparison of peak retention time and area of reference standards.

### Statistical analysis

Experiments were conducted in triplicate and values represent mean of *n* = 3± standard error of the mean (SEM) or standard deviation (SD) as indicated. *p* values for statistical significance were determined by two-way ANOVA with Tukey *post hoc* analysis for co-treatments and, one-way ANOVA with Dunnett’s multiple comparison or Tukey *post hoc* analysis for single treatments, *p* ≤ 0.05 (*), *p* ≤ 0.01 (**), *p* ≤ 0.001 (***), *p* ≤ 0.0001 (****). GraphPad Prism software version 7.01 was used to generate figures and perform statistical analysis (GraphPad Software, San Diego, CA, United States).

## Results

### HMC3 cells express some receptors typical of microglia

**FIGURE 1:**
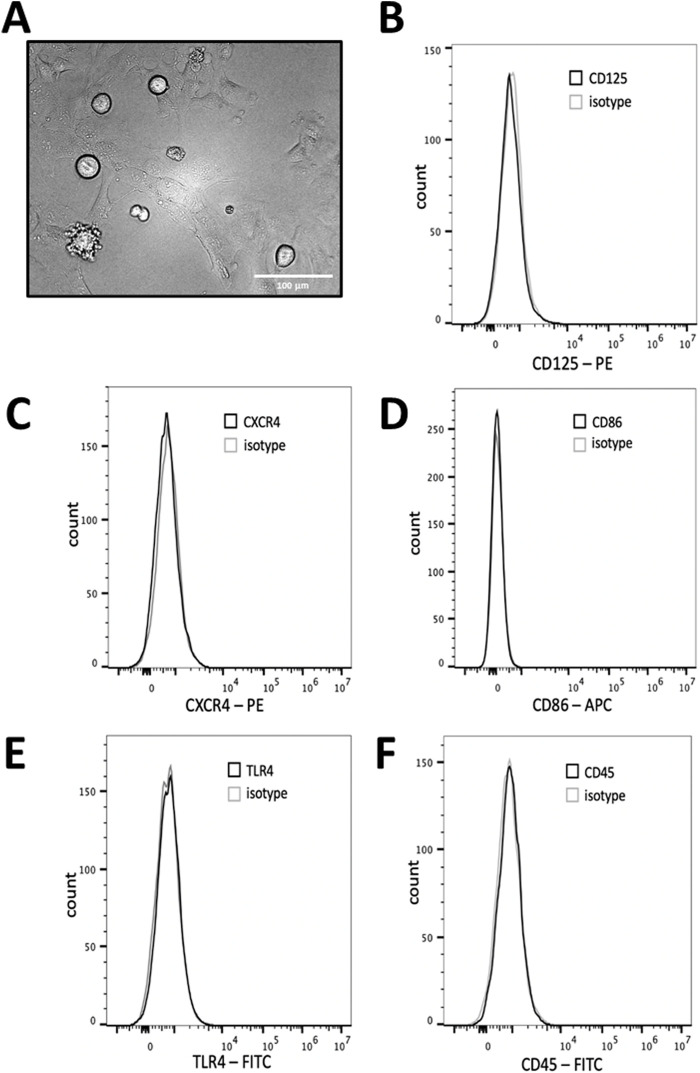
HMC3 cell morphology and biomarker expression. **(A)** HMC3 imaged *via* phase contrast microscopy at ×20 magnification. HMC3 were plated and left, unstimulated, for 24 h and analyzed for expression of **(B)** CD125, **(C)** CXCR4, **(D)** CD86, **(E)** TLR4, or **(F)** CD45 and analyzed *via* flow cytometry. Data shown is representative of three independent experiments (*n* = 3).

Since HMC3 are considered an imprecise model of primary human microglia (He et al., 2021; dello Russo et al., 2018), we characterized some of their common features. Light microscopic analysis indicated that some of the cells had the expected globular morphology while most of the cells were elongated with thick processes (Figure 1A). We measured HMC3 expression of CD125, CXCR4, CD86, TLR4, and CD45, by flow cytometry. Non-stimulated HMC3 showed little to no expression of any of these receptors (Figures 1B–F). These data support previous data that show that HMC3 express few microglial surface receptors in the absence of stimuli (Li et al., 2009; Rai et al., 2020). Our data contribute to the characterization of HMC3 as a model of microglial research by further indicating that, in the absence of stimulation, HMC3 express few microglial receptors.

### Activated HMC3 produce IL-8 and IL-6

**FIGURE 2:**
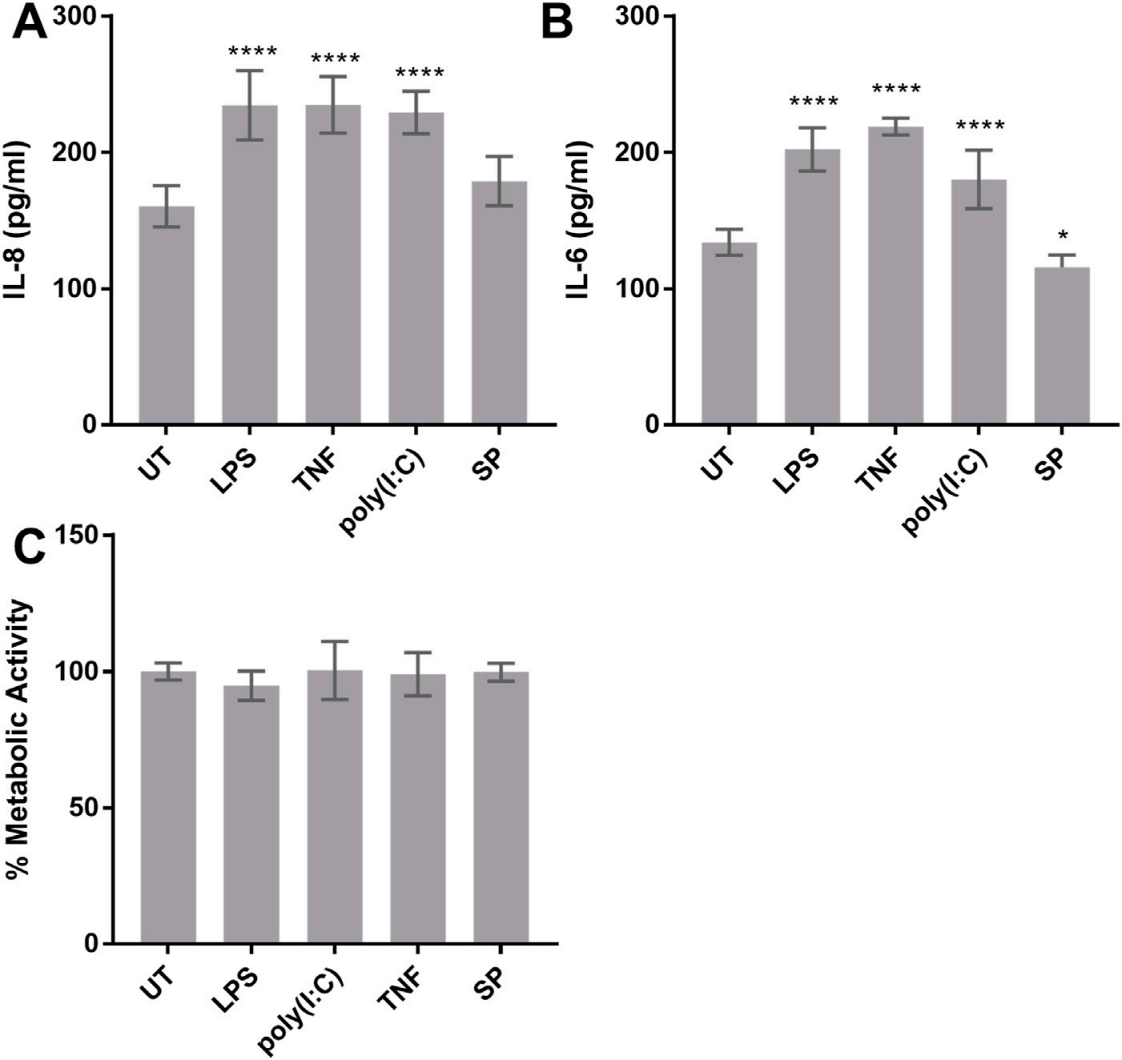
Cytokine release and metabolic activity of HMC3. **(A, B)** HMC3 were stimulated with 1 µg/mL LPS, 0.25 µg/mL TNF, 0.5 µg/mL poly(I:C), 0.5 µg/mL substance P (SP), or left untreated (UT) for 24 h and IL-8 and IL-6 production was measured by ELISA. **(C)** HMC3 were activated as in **(A)** and metabolic activity was determined by reduction of XTT. Data are presented as mean ± SEM for *n* = 3. Statistical significance was calculated using one-way ANOVA with Dunnett’s multiple comparison pot-hoc analysis relative to untreated (UT): *p* ≤ 0.05 (*), *p* ≤ 0.0001 (****).

To better understand the response of HMC3 to common neuroinflammatory stimuli, we activated HMC3 with LPS, TNF, poly(I:C) and substance P and measured their release of IL-8 and IL-6 (Figures 2A, B). HMC3 response to poly(I:C) and substance P had not been previously described. HMC3 constitutively released significant amounts of both IL-6 and IL-8, and when they were activated with LPS, TNF and poly(I:C) they produced approximately 30% more of both mediators compared to untreated cells. Substance P did not significantly increase IL-8 release and significantly reduced the basal amount of IL-6 released from HMC3.

Since the metabolic rate of microglia is often increased after activation, we measured their metabolic rate using a standard formazan reduction assay by which XTT (2,3-bis(2-methoxy-4-nitro-5-sulfophenyl)-5-carboxanilide-2H-tetrazolium) is reduced by mitochondrial produced NADH to a soluble orange-yellow formazan salt. Activation of HMC3 with LPS, TNF, poly(I:C) or substance P did not alter the metabolic rate of HMC3 cells (Figure 2C).

### LPS and poly(I:C) do not significantly upregulate receptor expression

**FIGURE 3:**
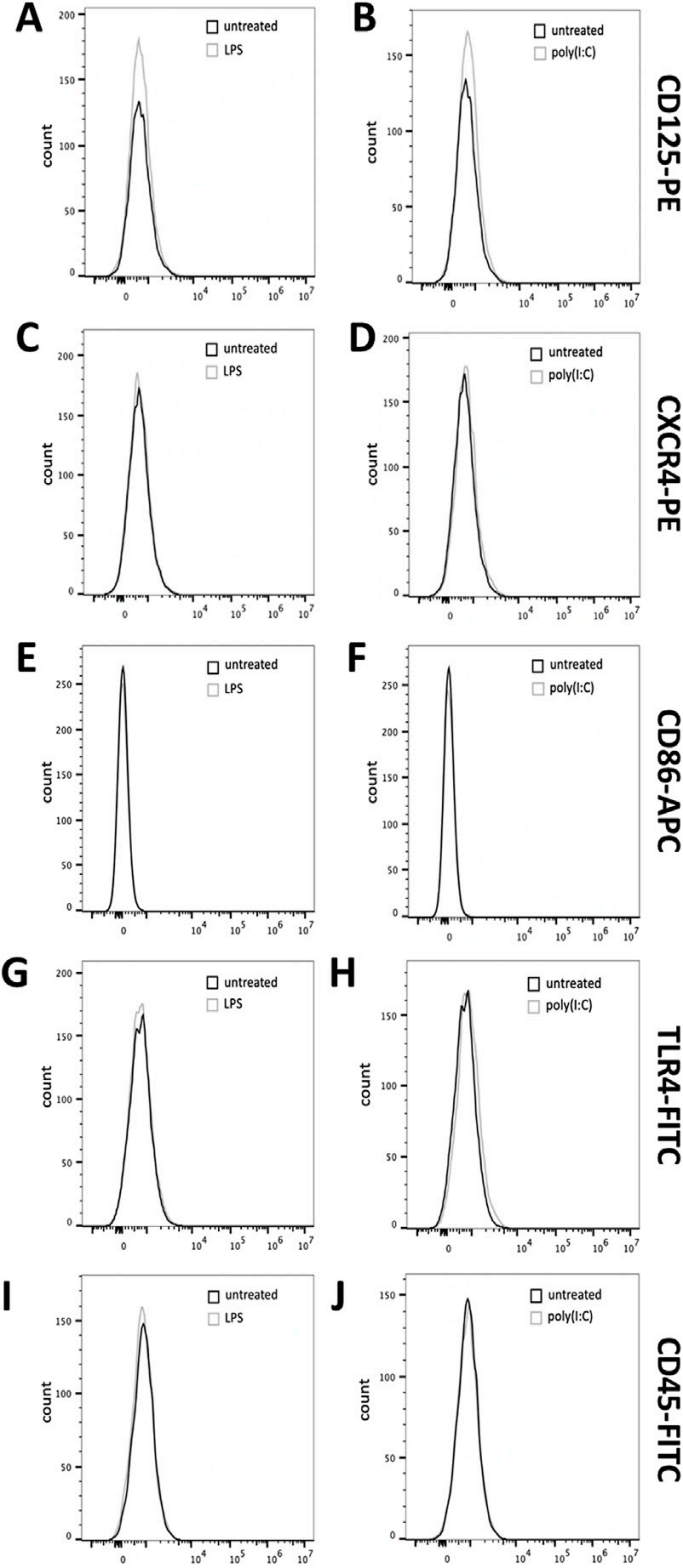
HMC3 microglial receptor expression after stimulation with LPS or poly(I:C). HMC3 were stimulated with 1 μg/mL LPS or 0.5 μg/mL poly(I:C) for 24 h, and expression of **(A, B)** CD125-PE, **(C, D)** CXCR4-PE, **(E, F)** CD86-APC, **(G, H)** TLR4-FITC or **(I, J)** CD45-FITC was analyzed by flow cytometry. Data shown is representative of three independent experiments (*n* = 3).

Since LPS and poly(I:C) differentially modulate primary microglia activation (He et al., 2021), we tested their effect on HMC3 receptor expression (Figure 3). We focused on poly(I:C) since poly(I:C) effects on HMC3 had not been previously described. We chose LPS as a positive control since LPS effects on HMC3 have been extensively described in the literature. Our data indicates that 24 h exposure to LPS or poly(I:C) do not alter the expression of microglial surface receptors CD125, CXCR4, CD86, TLR4, and CD45.

### AME-1 activates HMC3 production of several cytokines

**FIGURE 4:**
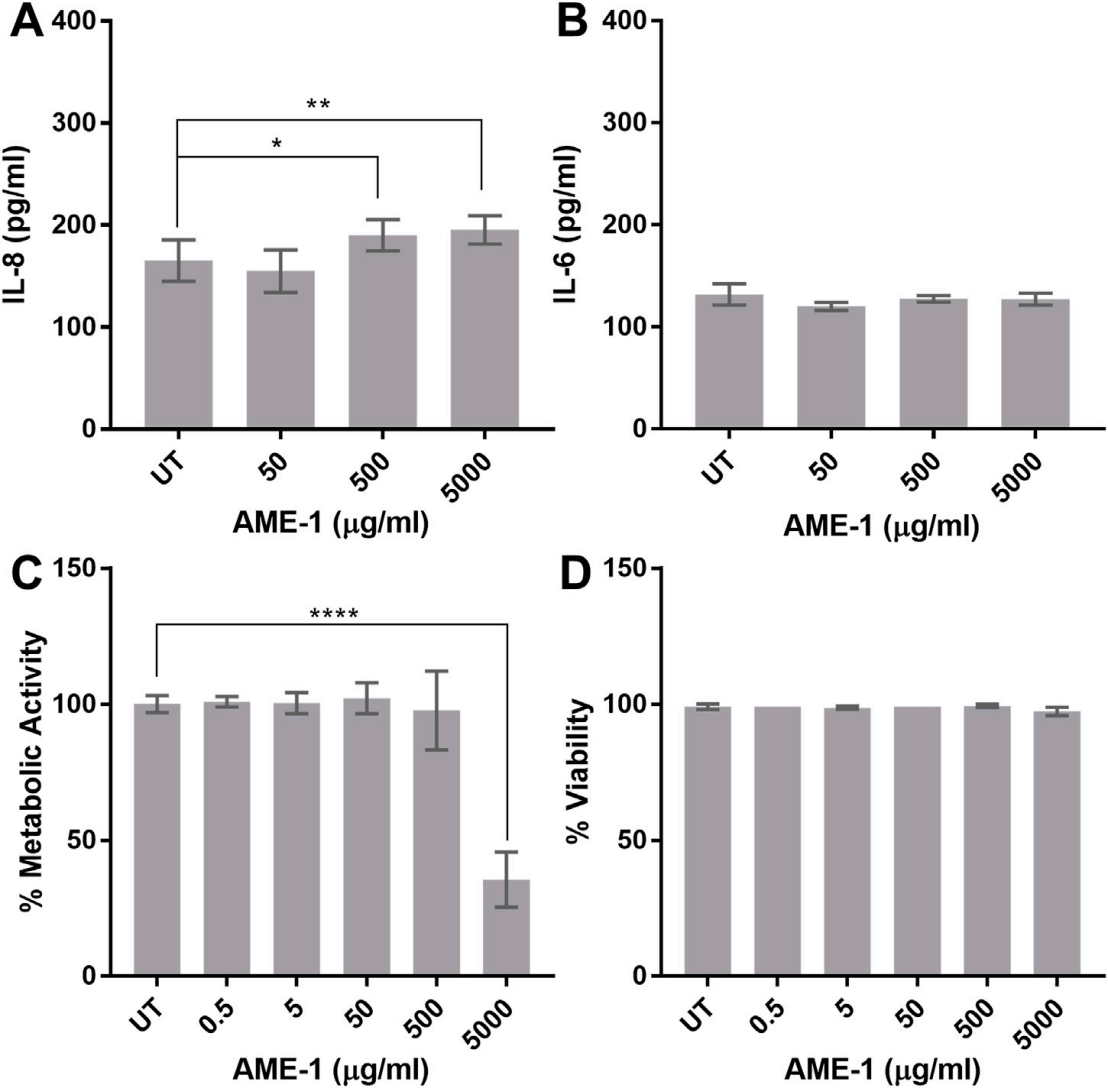
AME-1 effect on IL-8 and IL-6 production and metabolic activity. **(A, B)** HMC3 were treated with AME-1 (50–5,000 µg/mL) or left untreated (UT) for 24 h and IL-8 and IL-6 production was measured by ELISA. HMC3 were treated as in **(A)** and metabolic activity and viability was analyzed *via* **(C)** XTT and **(D)** trypan blue exclusion assay. Data presented as mean ± SEM for *n* = 3. Statistical significance was calculated using one-way ANOVA with Dunnett’s multiple comparison pot-hoc analysis relative to untreated (UT): *p* ≤ 0.05 (*), *p* ≤ 0.01 (**), *p* ≤ 0.0001 (****).

We next determined the effect of *A. muscaria* extract (AME-1) on HMC3 production of cytokines using two different approaches: ELISA and electrochemiluminescent multiplex analysis. ELISA analysis indicates that at higher concentrations, AME-1 significantly increased basal IL-8 production but did not significantly increase IL-6 production, even at extremely high concentrations of 5,000 µg/mL (Figures 4A, B). AME-1 also did not affect HMC3 metabolic activity (Figure 4C), although the very highest concentration of 5,000 µg/mL decreased their metabolic activity by approximately 60% compared to the untreated control (UT). AME-1 did not affect cell viability after 24 h at even the highest concentration of 5,000 µg/mL (Figure 4D) which indicates that although the metabolic rate of cells treated with 5,000 μg/mL was significantly reduced, the cells were still viable and had not undergone necrosis.

**FIGURE 5:**
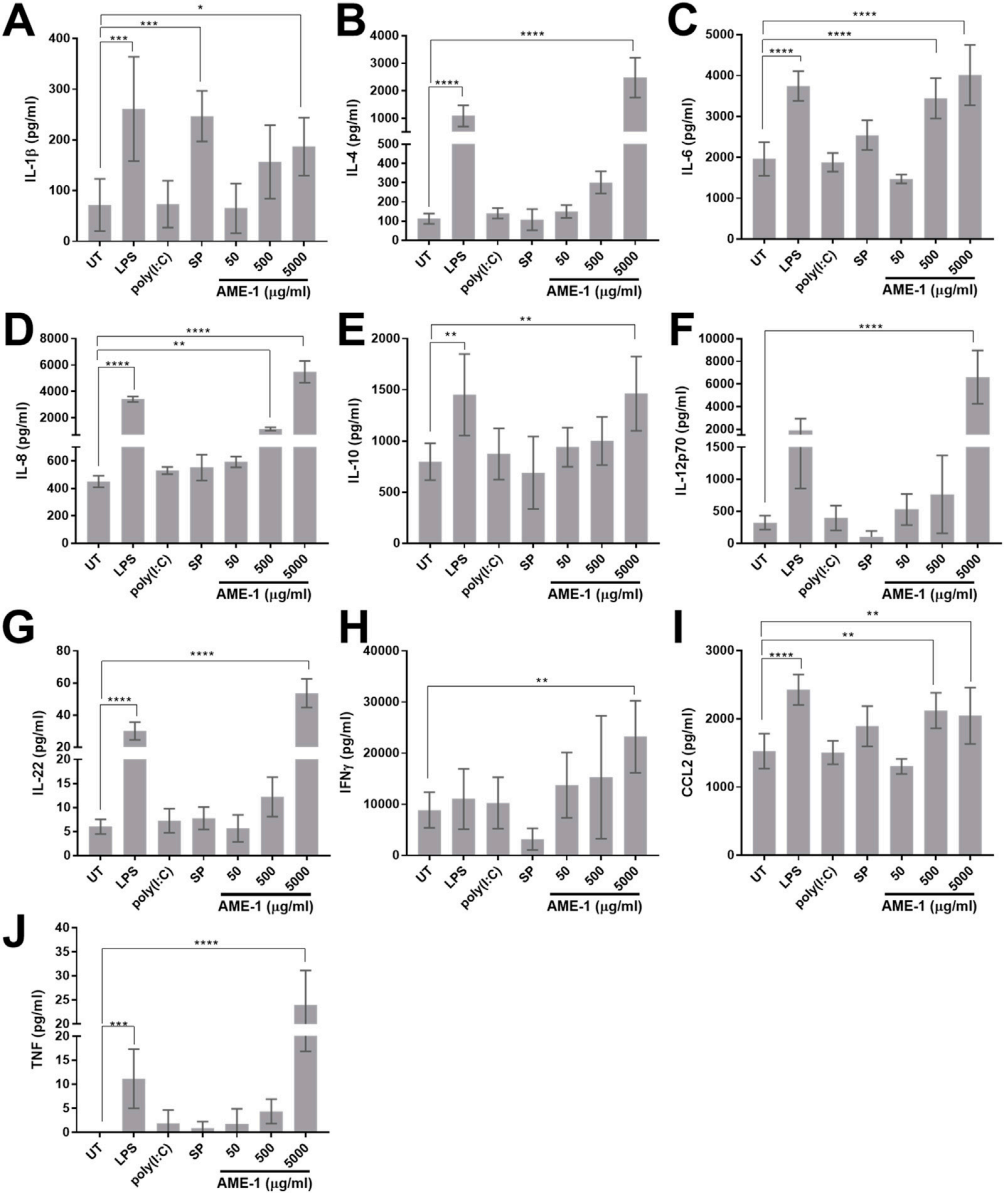
AME-1 significantly increases production of various cytokines. HMC3 were stimulated with 1 µg/mL LPS, 0.5 µg/mL poly(I:C), 0.5 µg/mL substance P (SP), or left untreated (UT) for 24 h. Cell free supernatants were collected and analyzed *via* electrochemiluminsescent analysis for **(A)** IL-1β, **(B)** IL-4, **(C)** IL-6, **(D)** IL-8, **(E)** IL-10, **(F)** IL-12p70, **(G)** IL-22, **(H)** IFN-γ, **(I)** CCL2 and, **(J)** TNF release. Data presented as mean ± SEM for *n* = 3. Statistical significance was calculated using one-way ANOVA with Tukey *post hoc* analysis: *p* ≤ 0.05 (*), *p* ≤ 0.01 (**), *p* ≤ 0.001 (***), *p* ≤ 0.0001 (****).

Electrochemiluminescent multiplex analysis showed that AME-1 significantly induced the production of IL-1β, IL-4, IL-6, IL-8, IL-10, IL-12p70, IL-22, IFN-γ, CCL2 and TNF (Figure 5). HMC3 did not significantly release IL-12p70 or IFN-γ upon LPS stimulation (Figures 5F, H). Poly(I:C) and substance P did not significantly induce the release of any of the mediators that were measured.

### AME-1 upregulates expression of receptors

**FIGURE 6:**
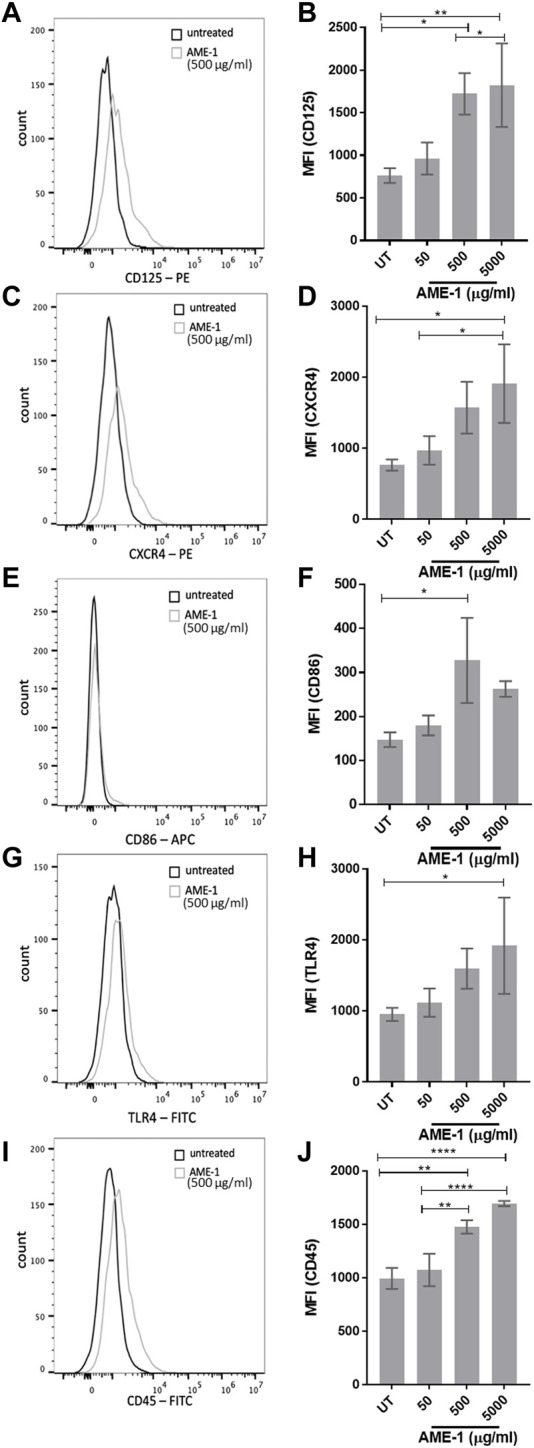
AME-1 significantly increases expression of CD125, CXCR4, CD86, TLR4, and CD45. MFI histograms and corresponding bar graphs of HMC3 treated with 50–5,000 μg/mL of AME-1 for 24 h,labelled with **(A, B)** PE-CD125, **(C, D)** PE-CXCR4, **(E, F)** APCCD86, **(G, H)** FITC-TLR4 or **(I, J)** FITC-CD45 and analyzed *via* flow cytometry. Data shown in flow cytometry histograms is representative of three independent experiments (*n* = 3). Mean fluorescence intensity (MFI) values for all three experiments are presented as mean ± SEM forn = 3. Statistical significance was calculated using one-way ANOVA with Tukey post hoc analysis, *p* ≤ 0.05 (*), *p* ≤ 0.01 (**), *p* ≤ 0.0001 (****).

We next determined the effect of AME-1 on receptor expression by HMC3 (Figures 6A–J). AME-1 increased the expression of CD125 CXCR4, TLR4, CD86 and CD45 when compared to untreated cells. MFI analysis of three independent experiments confirmed that AME-1 significantly and reproducibly increased expression of CD125, CXCR4, CD86, TLR4 and CD45. AME-1 increased expression of CD125, CXCR4, TLR4 and CD45 in a concentration-dependent manner, with the highest AME-1 concentration (5,000 μg/mL) showing the highest increase in receptor expression. AME-1 upregulation of CD86 was only significant at the 500 µg/mL concentration. Although AME-1 appeared to also increase expression of TLR4, this was not statistically significant in this study.

### AME-1 effects on stimulated HMC3

**FIGURE 7:**
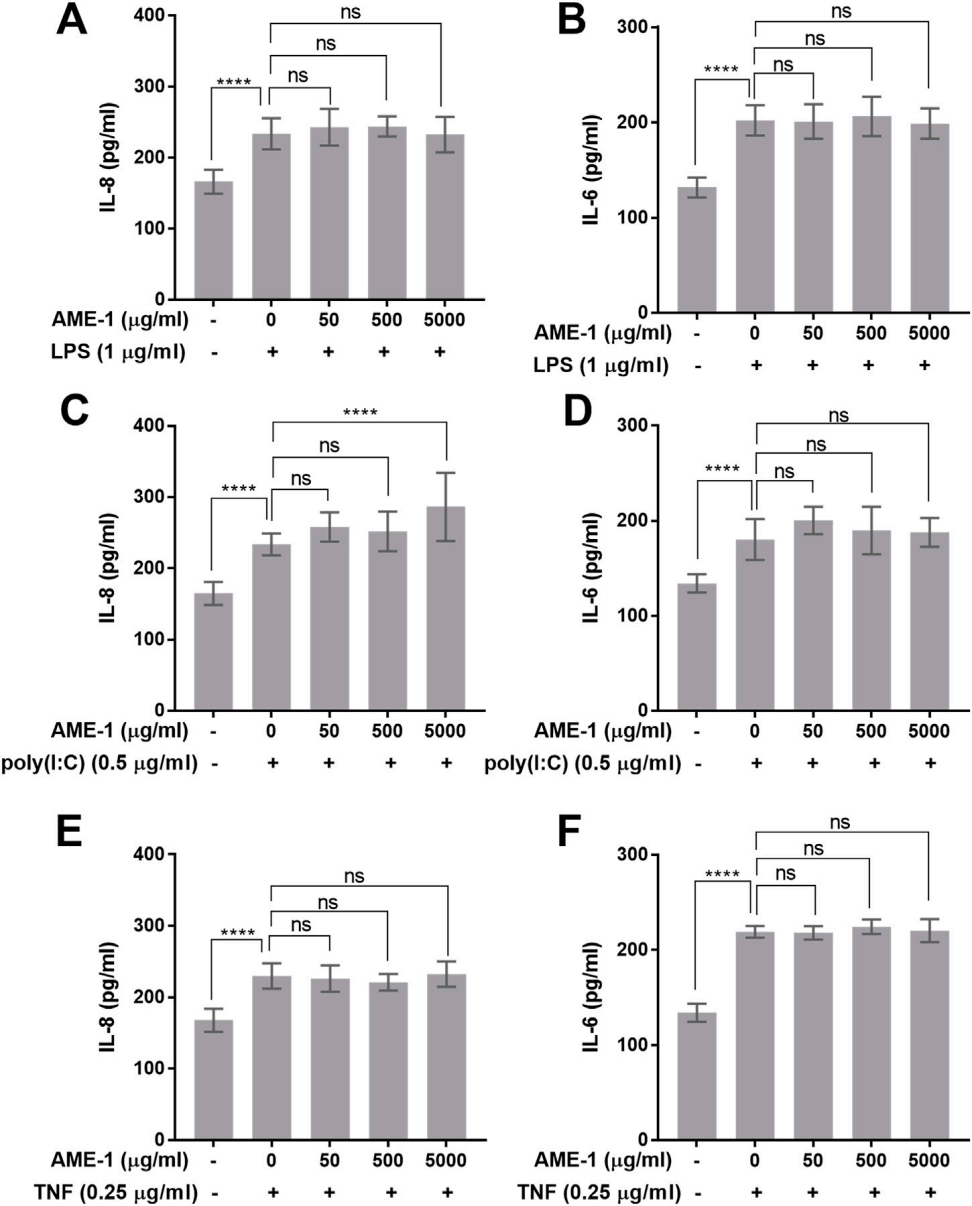
AME-1 potentiates poly(I:C)-induced production of IL-8. HMC3 were treated with AME-1 (50–5,000 μg/mL) or left untreated for 3 h and stimulated with 1 μg/mL LPS, 0.25 μg/mL TNF, or 0.5 μg/mL poly(I:C) for 24 h. **(A, B)** AME-1 has no effect on LPS-induced production of IL-8 or IL-6. **(C, D)** AME-1 potentiates poly(I:C)-induced production of IL-8 but has no effect on IL-6. **(E, F)** AME-1 has no effect on TNF-induced production of IL-8 or IL-6. Data presented as mean ± SEM for *n* = 3. Statistical significance was calculated using Two-Way ANOVA with Tukey post hoc analysis: *p* ≤ 0.0001 (****).

Next, the combined effect of AME-1 and other stimulants on HMC3 was evaluated. HMC3 were treated with AME-1 and then stimulated with LPS, poly(I:C), or TNF and their production of IL-8 and IL-6 was measured. AME-1 had no effect on LPS-induced production of either IL-8 or IL-6 (Figures 7A, B). However, AME-1 potentiated poly(I:C)-induced production of IL-8 (Figure 7C). AME-1 did not enhance poly(I:C)-induced IL-6 production (Figure 7D). AME-1 had no effect on TNF-induced production of IL-8 or IL-6 (Figures 7E, F).

### AME-1 effects on the expression of dsRNA sensing proteins

**FIGURE 8:**
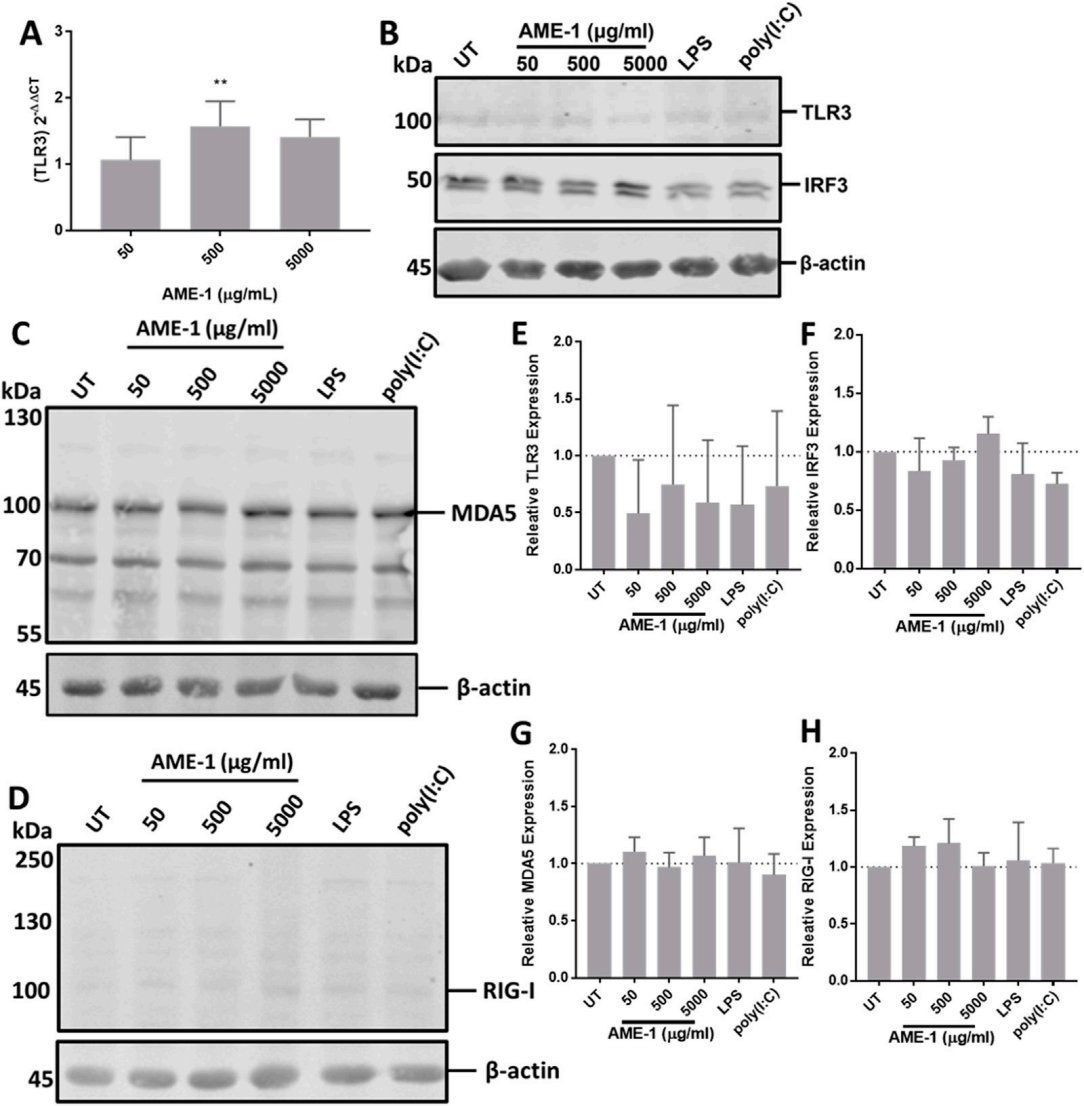
AME-1 upregulates TLR3 expression. **(A)** HMC3 cells were treated with AME-1 (50–5,000 μg/mL) for 3 h, RNA was extracted and analyzed *via* RT-qPCR. Results are presented as fold-gene expression relative to untreated cells and statistics were calculated using One-Way ANOVA with Dunnett’s multiple comparison *post hoc* analysis relative to fold-change in GAPDH expression: *p* ≤ 0.01 (**). Data presented as mean ± SEM for *n* = 3. HMC3 were treated with AME-1 (50–5,000 µg/mL), LPS (1 µg/m) or poly(I:C) (0.5 µg/mL) for 24 h, the cells were lysed and analyzed *via* Western blot. **(B)** TLR3 and IRF3 protein expression and **(E, F)** corresponding densitometry analysis. **(C, D)** MDA5 and RIG-I protein expression and **(G, H)** corresponding densitometry analysis. Densitometry analysis was performed on three blots on the indicated bands and statistical significance was calculated using one-way ANOVA with Tukey *post hoc* analysis.

Since poly(I:C) is a potent ligand of TLR3, we next measured the effect of AME-1 on TLR3 expression by HMC3. AME-1 had a significant effect on the expression of TLR3 mRNA (Figure 8A) by HMC3 cells, although this effect was modest (approximately 2-fold increase with 500 μg/mL of AME-1). The highest concentration of AME-1 of 5,000 μg/mL did not modify expression of TLR3 mRNA, suggesting that this effect reaches saturation at these extremely high concentrations.

Western blot analysis indicated that AME-1 had no significant effect on TLR3 protein expression or expression of the interferon regulatory transcription factor (IRF3), even at the highest concentration of 5,000 μg/mL (Figures 8B, E, F). RIG-I and MDA5 are other receptors that can bind and sense dsRNA (Brisse and Ly, 2019) and thus we evaluated their expression following AME-1 treatment. AME-1 appeared to upregulate MDA5 expression (Figure 8C) and RIG-I (Figure 8D), although this effect was not statistically significant over three experiments (Figures 8G, H).

### AME-1 contains significant amounts of trehalose

**FIGURE 9:**
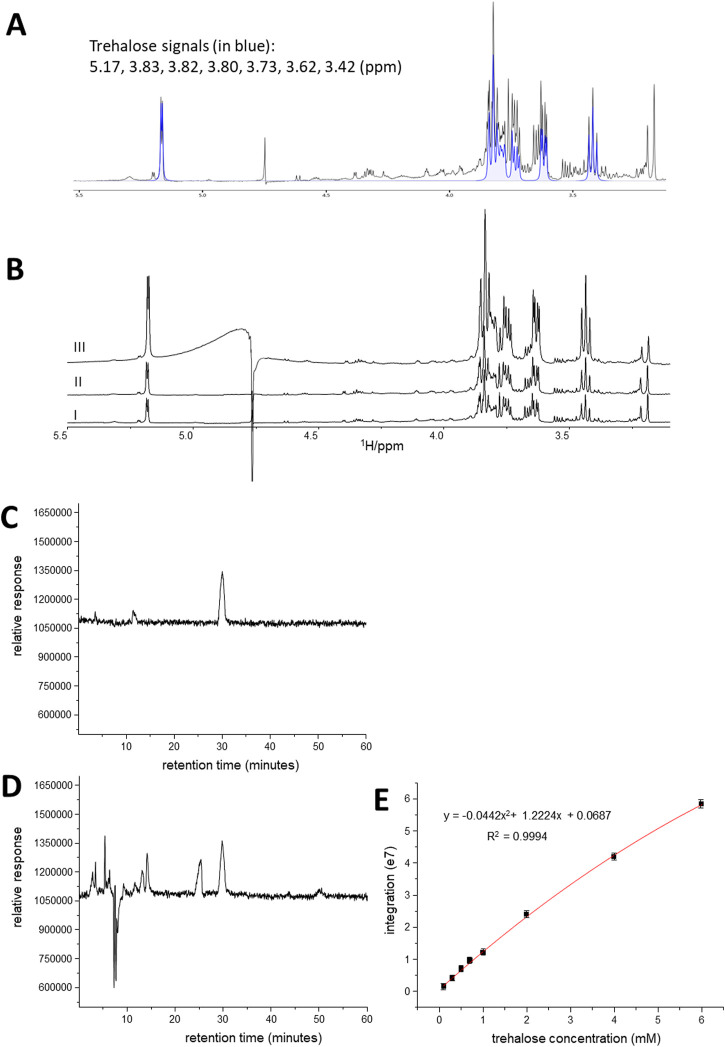
Analysis of AME-1 by NMR and HPLC. **(A)** Expanded region of ^1^H NMR of AME-1 highlighting the signals of trehalose (in blue color). (B) ^1^H NMR of AME-1 before (I) and after trehalose spiking (II: with 0.7 mM trehalose; III: with 4 mM trehalose). The intensity of signals centered at 5.17, 3.83, 3.80, 3.73, 3.62, and 3.42 ppm was increased when trehalose was added. **(C–E)** HPLC-MS chromatograms of trehalose standard **(C)** and 100X dilute AME-1 **(D)** showing the separation of trehalose from other metabolites. The mass spectrometer was operated in scan mode **(C, D)**. **(E)** Trehalose calibration curve. Standards containing varying concentrations of trehalose were injected onto the LC-MS with SIM mode. Data presented as mean ± SD for *n* = 4.

To get a better understanding of the components of AME-1, a metabolomic profile of the extract was achieved by combining NMR spectroscopy with metabolites identification and quantification software, i.e., ChenomX NMR suite. Some typical metabolites were identified (Table 1) and several signals could not be assigned to a particular metabolite due to the limitations of the database. The strongest signals in the aliphatic region of ^1^H NMR spectrum were due to trehalose which was the most abundant metabolite found in the extract (Figure 9A). 4-Aminobutyrate (GABA) was also observed with a relatively low content level in the proton NMR spectrum of AME-1. In the aromatic region, uridine, cytosine, ADP, tyrosine, phenylalnine, guanosine, and nicotinate were detected. The identity of trehalose was further confirmed by the NMR spiking experiments (Figure 9B). The concentration of trehalose in the 100X dilute solution is 1.38 mM as revealed by the ^1^H NMR analysis.

**TABLE 1:** Metabolite composition of AME-1 as analyzed by NMR.

	Compound name	Concentration (mM)
1	2-Aminoadipate	0.12949
2	2-Hydroxy-3-methylvalerate	0.12315
3	2-Hydroxybutyrate	0.08419
4	2-Hydroxyglutarate	0.12585
S	2-Hydroxyvalerate	0.04945
6	2-Methylgiutarate	0.03953
7	2-Octenoate	0.11389
8	2-Oxocaproate	0.03706
9	2-0xosilutarate	0.13334
10	2-Phenylpropionate	0.02588
11	3,5-Dibromotyrosine	0.04653
12	3-Aminoisobutyrate	0.06488
13	3-Hydroxy-3-methyiglutarate	0.06143
14	3-Methylgiutarate	0.04506
15	4-Aminobutyrate	0.02188
16	Acetate	0.02562
17	Adenosine	0.01331
18	ADP	0.0351
19	Arabinitol	0.10428
20	Arginine	0.10289
21	Aspartate	0.07789
22	Azelate	0.04827
23	Betaine	0.0201
24	Biotin	0.05926
25	Butyrate	0.04999
26	Carnitine	0.09769
27	cis,cis-Muconate	0.02202
28	Citraconate	0.02804
29	Citrate	0.02914
30	Cytosine	0.02576
31	dTTP	0.02821
32	Formate	0.02408
33	Fucose	0.17544
34	Galactarate	0.03687
35	Glucarate	0.09589
36	Glycerol	0.11244
37	Guanidoacetate	0.48254
38	Guanosine	0.03392
39	Histidine	0.01991
40	Hydroxyacetone	0.04515
41	InosIne	0.01061
42	Isocitrate	0.59453
43	Isoleuclne	0.06338
44	Lactate	0.09885
45	Leucine	0.04843
46	Lysine	0.08915
47	malate	0.02791
48	Malonate	0.02636
49	MethlonIne	0.03011
50	N,N-DImethylformamIde	0.07896
51	N-Acetylcysteine	0.10239
52	Nicotinate	0.03333
53	Ornithine	0.02167
54	Phenylacetate	0.01638
55	PhenylalanIne	0.06351
56	Propylene glycol	0.03872
57	Succinate	0.02113
58	Syringate	0.00713
59	Tartrate	0.12868
60	Taurine	0.16596
61	Trehalose	1.37787
62	Tyrosine	0.03146
63	Uddlne	0.05346
64	Valine	0.08462
65	8-Alanine	0.08225

Although the trehalose in AME-1 has been identified and quantified, we wanted to confirm using HPLC-MS (Figures 9C–E). Therefore, we used a straightforward HPLC-MS based assay for the detection and quantification of trehalose in AME-1, demonstrating that trehalose could be separated from other metabolites and detected using a single quadrupole mass spectrometer with electrospray ionization (ESI-MS). In the positive scan mode, a mass range of 50—1,000 was chosen to cover the m/z value of 360 corresponding to the [M + NH_4_^+^] parent ion. As can be seen, the retention time for trehalose is 30.1 min (Figure 9C), and the trehalose signal was separated from other metabolite signals in the AME-1 extract (Figure 9D).

Having determined that trehalose can be easily separated and detected using this HPLC-MS method, a calibration curve for trehalose was constructed (Figure 9E). In these experiments, the mass spectrometer was operated in single ion monitoring (SIM) mode in which an m/z of 360 due to [M + NH_4_^+^] parent ion was used. The relative response of SIM was used to plot the standard curve. The standard curves are best fit by a single polynomial. The polynomial fits produce *R* ^2^ value of greater than 0.998 indicating low accuracy errors.

To determine the concentration of trehalose in the AME-1 by HPLC-MS, 100x diluted AME-1 in water was used for this experiment. The peak for trehalose in SIM mode was detected at the expected retention time for 100x diluted AME-1 sample. Based on the standard curve, the trehalose concentration in the 100x diluted AME-1 was calculated to be 0.91 ± 0.06 mM (*n* = 4). These data demonstrate, for the first time, that trehalose contained within the AME-1 extract can be successfully detected and quantified using HPLC-MS.

It is noteworthy that the trehalose concentration in 100X dilute AME-1 was determined by NMR spectroscopy and HPLC-MS. HPLC-MS shows a relatively low trehalose concentration compared to NMR analysis. This fact may be due to the complex composition of AME-1, and the NMR signals were overlapped in the spectrum. The strong water signal in AME-1 could cause the baseline of the NMR spectrum to become distorted, and the relative signal intensities due to metabolites may have therefore been over-estimated. Therefore, the trehalose concentration in the original AME-1 without dilution would be 0.091 M based on the HPLC-MS.

### Trehalose potentiates poly(I:C) activation of HMC3

**FIGURE 10:**
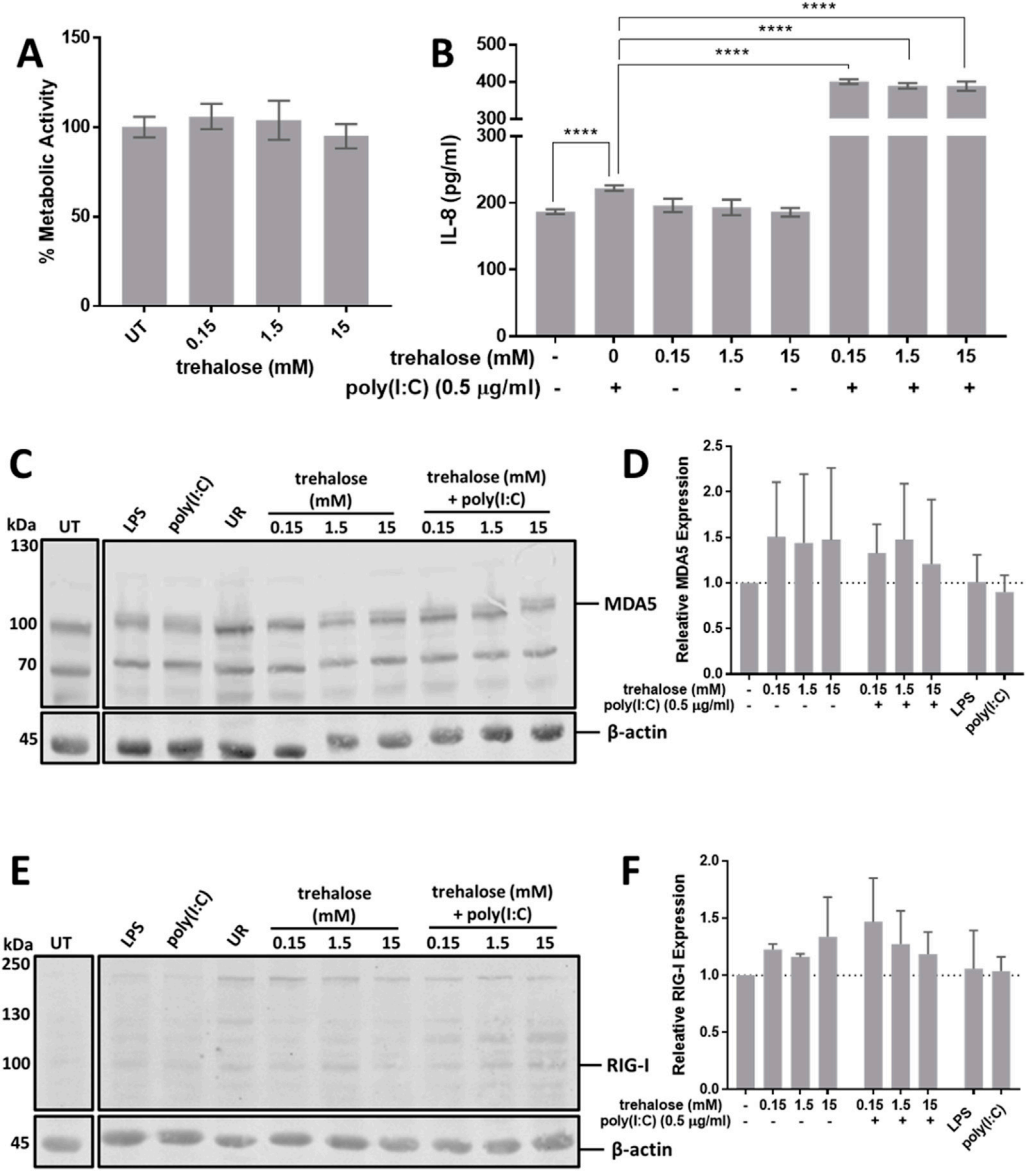
Trehalose potentiates poly(I:C) mediated activation of HMC3. **(A)** HMC3 were treated with 0.15–15 mM of trehalose for 24 h and metabolic activity was assessed *via* XTT assay. **(B)** HMC3 were left untreated (UT) or treated with 0.15–15 mMof trehalose for 3 h and subsequently activated with 0.5 μg/mLof poly(I:C) for 24 h and **(B)** cell free supernatants were measured for IL-8 *via* ELISA. Data presented as mean ± SEM for *n* = 3. Statistical significance was calculated using two-way ANOVA with Tukey post hoc analysis: *p* ≤ 0.0001 (****). **(C, E)** Cell lysates were analyzed for MDA5 and RIGI expression *via* Western blot and **(D, F)** corresponding densitometry analysis was performed. Unrelated samples are annotated as UR. Western blot images are representative of *n* = 3. Densitometry data presented as mean ± SEM for *n* = 3 and statistical analysis was calculated using one-way ANOVA with Tukey post hoc analysis.

**FIGURE 11:**
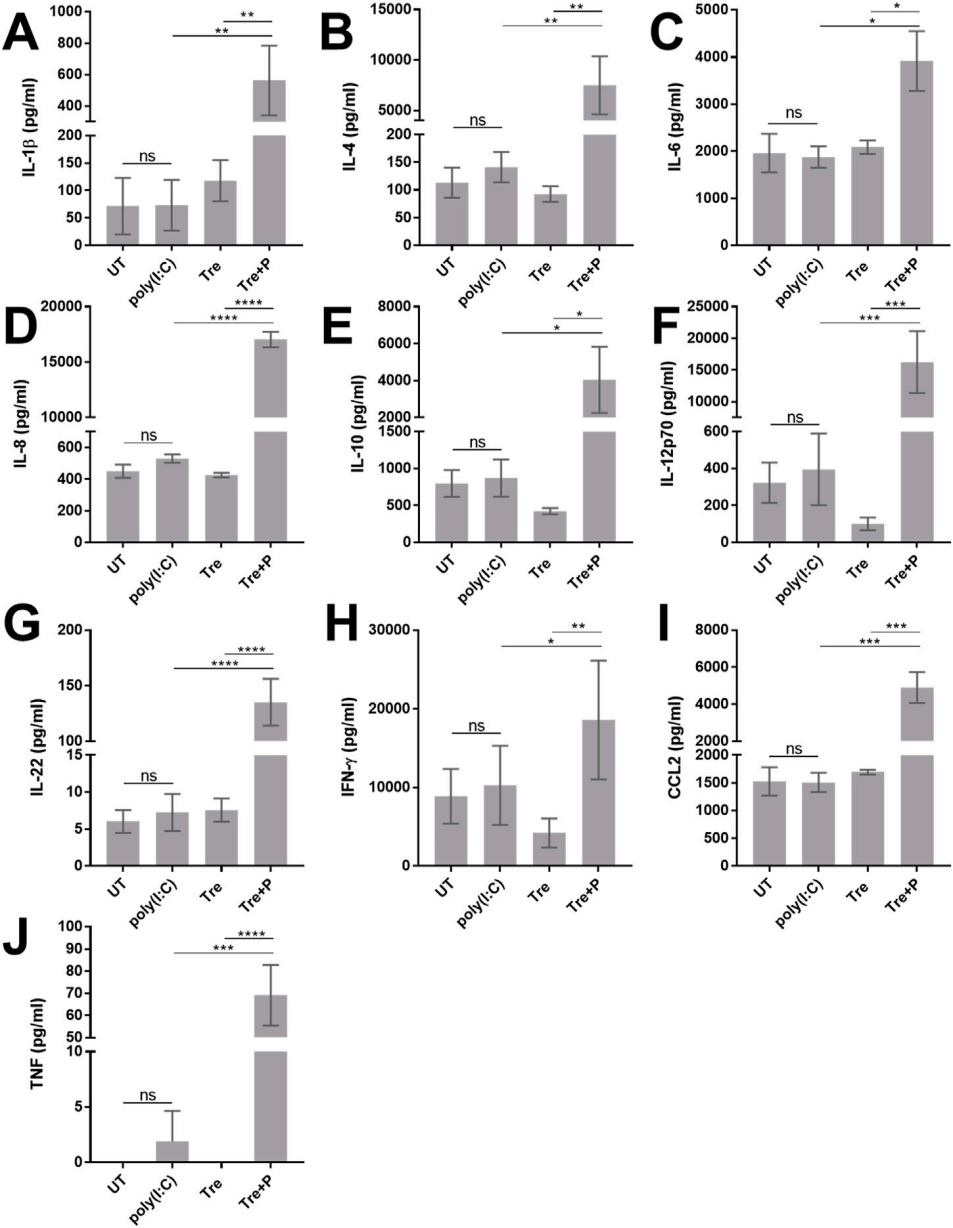
Trehalose potentiates poly(I:C)-mediated release of various cytokines. HMC3 were left untreated (UT) or treated with 0.15 mM trehalose (Tre) for 3 h and subsequently stimulated with 0.5 μg/mL poly(I:C) for 24 h (Tre + P). For comparison/controls, HMC3 were treated with 0.5 μg/mL poly(I:C) for 24 h or 0.15 mM trehalose (Tre) for 24 h. Cell free supernatants were collected and analyzed *via* electrochemiluminescent multiplex analysis for **(A)** IL-1β, **(B)** IL-4, **(C)** IL-6, **(D)** IL-8, **(E)** IL-10, **(F)** IL-12p70, **(G)** IL-22, **(H)** IFN-γ, **(I)** CCL2 and, **(J)** TNF. Data are presented as mean ± SEM for *n* = 3. Statistical significance was calculated using two-way ANOVA with Tukey post hoc analysis: *p* > 0.05 (ns), *p* ≤ 0.05 (*), *p* ≤ 0.01 (**), *p* ≤ 0.001 (***), *p* ≤ 0.0001 (****).

Since AME-1 contained high levels of trehalose, the effect of trehalose on HMC3 metabolic activity, IL-8 production and receptor expression was determined (Figure 10). Like AME-1, trehalose had no effect on HMC3 metabolic activity (Figure 10A), and potentiated poly(I:C)-activated production of IL-8 (Figure 10B). Trehalose potentiated poly(I:C)-activated production of IL-8 even at the lowest concentration of 0.15 μg/mL and increasing trehalose concentrations did not increase this effect. Trehalose also significantly potentiated poly(I:C) production of IL-1β, IL-4, IL-6, IL-8, IL-10, IL-12p70, IL-22, IFN-γ, CCL2, and TNF (Figures 11A–J). Trehalose treatment alone and trehalose followed by poly(I:C) stimulation did not significantly increase MDA5 expression (Figures 10C, D), although there was an increased trend in expression. Similarly, trehalose alone and combined treatment with poly(I:C) did not significantly affect RIG-I expression (Figures 10E, F).

### Trehalose effect on receptor expression by HMC3

**FIGURE 12:**
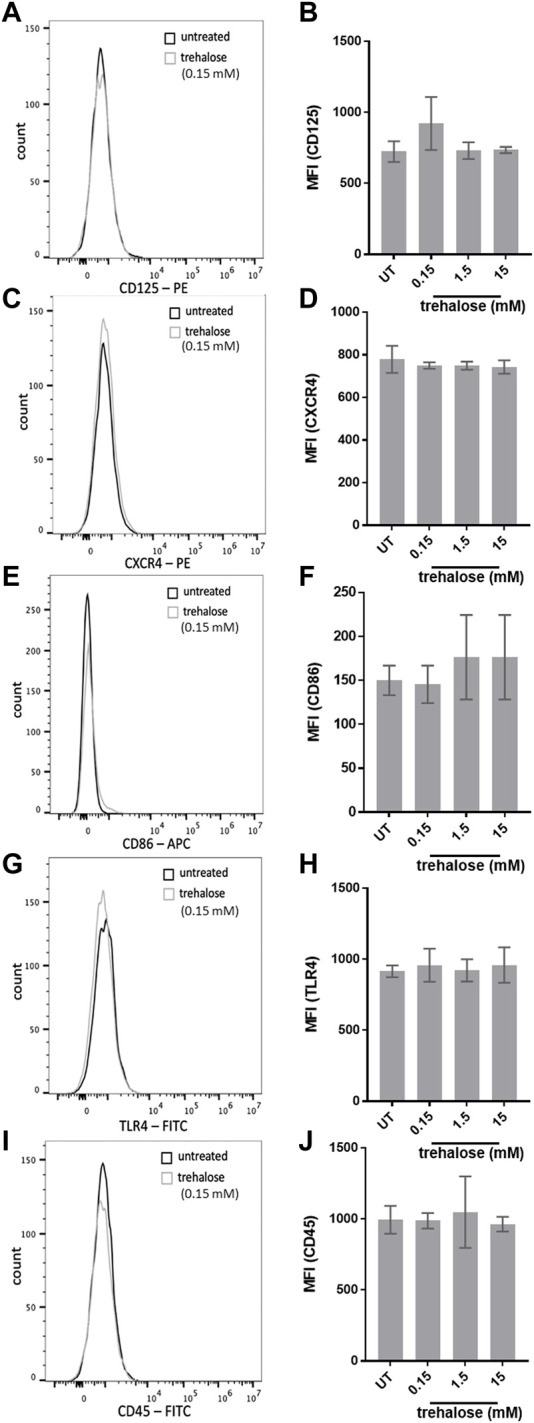
Effects of trehalose on HMC3 biomarker expression. Histograms and corresponding bar graphs of HMC3 left untreated or treated with 0.15–15 mM of trehalose for 24 h and expression of **(A, B)** CD125, **(C, D)** CXCR4, **(E, F)** CD86, **(G, H)** TLR4 or **(I, J)** CD45 was analyzed by flow cytometry. Data shown in flow cytometry histograms is representative of three independent experiments (*n* = 3). Mean fluorescence intensity (MFI) values for all three experiments are presented as mean ± SEM for *n* = 3. Statistical significance was calculated using one-way ANOVA with Tukey *post hoc* analysis.

Since AME-1 significantly increased receptor expression by HMC3 we investigated the effects of trehalose. Trehalose treatment did not alter surface expression of CD125, CXCR4, CD86, TLR4, or CD45 (Figures 12A–J) by HMC3 cells.

## Discussion

The effects of *A. muscaria* on neuroinflammatory cells, such as microglia, are unknown. In this study, the effect of *A. muscaria* extract (AME-1) on the human microglia cell line (HMC3) was examined. Initial characterization of HMC3 demonstrated that these cells are globular and project thick processes indicative of a microglial-like morphology. A screen of receptor expression indicated that HMC3 did not express many surface receptors, in keeping with much of the previously published studies which show that HMC3 do not express many receptors in their non-stimulated state (Li et al., 2009; Etemad et al., 2012). Our data verify previous work by Wang et al. and Rai et al. that have demonstrated that unstimulated HMC3 do not express CD86 (Wang et al., 2021a) or CD45 (Brisse and Ly, 2019). In primary human microglia, these receptors are considered to be markers of microglial stimulation (Böttcher et al., 2019). Interestingly, Rai et al. found that unstimulated HMC3 express detectable levels of CXCR4 (Brisse and Ly, 2019), but this may be due to different culture conditions as our cells were cultured in MEM with activated FBS rather than EMEM with heat-inactivated FBS. Interestingly, CXCR4 is typically found in unstimulated primary human microglia as a functional chemokine receptor (Albright et al., 1999). Our work was the first to test the expression of TLR4 in HMC3 microglia, and this microglial marker is typically found highly expressed in murine models (Parajuli et al., 2012), but not human models (Jack et al., 2005). Additionally, we were the first to test HMC3 expression of CD125 (IL-5Rα), a receptor for neutrophils in humans (Lawrence et al., 2022).

While non-stimulated HMC3 microglia do not express the receptors CD125, CXCR4, CD45, and TLR4, our data shows that stimulation with AME-1 increases expression of these receptors in a concentration-dependent manner. The literature indicates that stimulation of HMC3 with inflammatory signaling molecules such as IFN-γ or TNF sometimes modifies the expression of some microglial receptors such as CD11b (Li et al., 2009) and CD86 (Wang et al., 2021b). However, unlike previous reports suggesting that LPS may upregulate mRNA expression of receptors such as CD86 (Lu et al., 2021), we found that LPS and poly(I:C) did not upregulate surface receptor expression of CD86 or any of the receptors we tested *via* flow cytometry. This suggests that the stimulation of HMC3 occurs in a manner unique to AME-1. Notably, HMC3 did not alter expression of receptors when treated with the autophagic inducer, trehalose, indicating that other metabolites may be responsible for AME-1-induced HMC3 stimulation.

When stimulated with LPS, HMC3 have been shown by others to produce IL-6, IL-8, IL-12 and IFN-γ (Wang et al., 2021a). In our study, we examined cytokine production by HMC3 using two different approaches: ELISA and electrochemiluminescent multiplex analysis. The electochemiluminescent approach has a broader concentration range and is advertised as a more sensitive method of soluble analyte detection. Our electrochemiluminescent multiplex analysis showed that when activated with LPS, HMC3 produced IL-1β, IL-4, IL-6, IL-8, IL-10, IL-12p70, IL-22 and CCL2 but almost undetectable amounts of TNF and no IFN-γ above background levels. HMC3 activated with substance P produced only IL-1β, which suggests that this is a unique feature of activation through the neurokinin receptor on HMC3.

HMC3 activated with poly(I:C) produced very small amounts of any cytokine tested by electrochemiluminescent multiplex analysis. AME-1 treatment, however, increased expression of almost all cytokines compared to untreated cells. Our study is the first to demonstrate that HMC3 are capable of producing some of these cytokines and in response to these stimuli.

Microglia are resident macrophage-like cells of the central nervous system and are involved in immune responses driven by TLRs. Microglial responses to TLR3 appear to be tightly controlled through the cathepsin family. For example, primary murine microglia can be activated with poly(I:C), a TLR3 ligand, that increases their proinflammatory response though activation of cathepsin X (Pišlar et al., 2022), suggesting that this process is associated with inflammation-induced neurodegeneration. However, cathepsin H deficiency decreases hypoxia-ischemia-induced hippocampal atrophy in neonatal mice through attenuation of TLR3/IFN-β signaling, suggesting that this pathway may be neuroprotective (Ni et al., 2021). As such, upregulation of TLR3 by AME-1 may participate in this pathway, although whether the balance is shifted toward a pro-inflammatory neurodegenerative or neuroprotective pathway is still unknown and requires further experimentation. The effect of AME-1 on RIG-I and MDA5 was also determined since these two receptors are also important sensors of intracellular dsRNA. RIG-I is a cytosolic pattern recognition receptor that acts similarly to TL3 and activates the type-I interferon response. Interestingly, RIG-I mediates microglial responses to RNA and DNA viruses (Crill et al., 2015) and is upregulated in a mouse model of PTSD (Dong et al., 2020). MDA5 is also a cytosolic dsRNA receptor. MDA5 has been shown to play a role in autoimmune diseases such as inflammatory encephalopathy and Aicardi-Goutieres syndrome where microglia are activated and show increased phagocytosis (Onizawa et al., 2021). The precise expression pattern and function of these two dsRNA sensors is still poorly understood in microglia. Our analysis of RIG-I and MDA5 expression indicated that AME-1 might be increasing expression of these important receptors for dsRNA. However, statistically, these differences were not significant and further analysis, perhaps using more sensitive methods of protein detection are necessary to determine whether these receptors are involved in this process.

Our data suggest that the human HMC3 respond differently to LPS and poly(I:C) compared to mouse primary microglia. Whereas LPS causes mouse primary microglia to produce IL-6, poly(I:C) did not induce IL-6 or TNF production (even at 10 μg/mL poly(I:C) (He et al., 2021). Our ELSIA data shows that both LPS and poly(I:C) (0.5 μg/mL) activates HMC3 to produce both IL-6 and IL-8 but no detectable TNF. The electrochemiluminscent analysis indicates some TNF production by LPS stimulated HMC3, but it is very minimal. Since IL-8 is an important chemokine for proinflammatory cells in the brain during severe infection (Khaboushan et al., 2022; Wang et al., 2022) and some forms of neurodysfunction (Abdelaziz et al., 2022; de Koning et al., 2022), this may represent an important species difference between human and mouse microglial function or may reflect differences between primary microglia and this transformed cell line. Regardless, ours is the first report of IL-8 production by HMC3 in response to poly(I:C).

AME-1 had a significant effect on expression of TLR3 mRNA but not protein expression by HMC3, suggesting that AME-1’s potentiation of poly(I:C)-induced production of IL-8 occurs independently of changes in TLR3 protein expression. Similarly, AME-1 had no significant effect on RIG-I or MDA5 expression. To our knowledge, this is the first examination of *A. muscaria* effects on TLR3 expression in human microglia. However, there are some reports that TLR expression is altered in some forms of neuropathy including Alzheimer’s disease, Parkinson’s disease, and amyotrophic lateral sclerosis (Deleidi et al., 2010; Lee et al., 2015; Walker et al., 2018). Human primary microglia express several TLRs and produce IL-6 and TNF upon stimulation with TLR3 and TLR5 ligands (Mitterreiter et al., 2017) and changes in TLR expression are associated with damage to the nervous system (Fiebich et al., 2018), suggesting that changes in TLR may be a protective response. It is possible that our analysis was unable to detect changes in TLR3, RIG-I or MDA5 protein expression, and further analysis with different time points is required. Regardless, AME-1 modulation of TLR3 function may form the basis of a priming of the innate immune system in the brain.

It is possible that some of the other components of AME-1, such as the metabolites identified in our metabolomics screen in Table 1, are responsible for some of the effects on HMC3 observed in our study. One of the most abundant components of AME-1 is trehalose, an autophagy enhancer and regulator of neuroinflammation is produced by astrocytes in the rodent hippocampus (Martano et al., 2017) and may be involved in protection from neurotoxicity (Massenzio et al., 2018). Increasing autophagy in the spinal cord with trehalose, decreases inflammation and improves motor function deficits in a mouse model (Li et al., 2022). However, trehalose inhibits the production of IL-1β, IL-6, TNF and nitric oxide by LPS-stimulated BV-2 mouse microglial cells (He et al., 2014), suggesting that if trehalose is the compound responsible for the effects we observed on HMC3 cells, it is behaving differently in these human HMC3 cells compared to mouse models. Further experiments are necessary to precisely determine how trehalose may be involved in this dramatic induction of cytokine production in poly(I:C)-activated HMC3 cells.

Lastly, our data is the first report to show that the tachykinin, substance P activates HMC3 cells to produce proinflammatory cytokine IL-1β. Substance P is an important neuropeptide in mediating pain and neuropathy that binds and activates the neurokin-1 receptors (NK-1R). Although human primary microglia and astrocytes constitutively express full-length isoform of NK-1R and respond to substance P by producing IL-6 (Martano et al., 2017), HMC3 do not have this same response.

In this study, NMR profiling data shows that AME-1 contains several metabolites. At least 65 compounds were found in AME-1, and trehalose was the most abundant metabolite (highest concentration) in AME-1. We confirmed the presence of trehalose in AME-1 using HPLC-MS which is the standard analytical tool in the qualitative and quantitative analysis of potential pharmaceuticals and their related metabolites. The high sensitivity and accuracy of this approach is suitable for the rapid quantification of metabolites in extracts such as AME-1 in our study. Here both high field ^1^H NMR spectroscopy and HPLC-MS were used, and HPLC-MS provided complementary information to that of the NMR spectroscopy, showing that different analytical platforms and their combined use in studies is advantageous.

The NMR and HPLC analysis suggested that the high trehalose content of AME-1 could be responsible for some of the biological effects of AME-1. Certainly, trehalose significantly potentiated the poly(I:C)-induced production of several cytokines–demonstrating that the combination of trehalose + poly(I:C) is the most potent activator of HMC3 cytokine production described to-date. However, trehalose did not upregulate CD125, CXCR4, CD86, TLR4 or CD45 expression and trehalose treatment did not significantly modulate MDA5 or RIG-I expression, suggesting that trehalose activates an alternative pathway in HMC3, possibly through the autophagy process. Trehalose is a potent inducer of autophagy in many cell types, including microglia (Massenzio et al., 2018) and induction of autophagy can reduce neuroinflammation (Li et al., 2022), suggesting that the two processes may be linked. It is intriguing to speculate that autophagy maybe be playing an important role in HMC3 responses to poly(I:C) and induction of the inflammatory response. Recent data has shown that autophagy is involved in microglia induction of the important regulator of the Nod-like receptor protein 3 (NLRP3) inflammasome signaling pathway (Wang et al., 2023b), which is activated by a variety of TLR agonists including poly(I:C) and viruses. Autophagy alleviates sevoflurane-induced cognitive dysfunction in elderly rats (Zhou et al., 2023) and autophagy is an important component of HIV-1 and drug-induced neuroinflammaging (Sil et al., 2022). For this reason, small molecules that can modulate autophagy and the NLRP3 inflammasome have been proposed as treatments for neurodegenerative diseases such as Alzheimer’s disease (Jha et al., 2023). Further experimentation is necessary to determine if trehalose induces autophagic responses in microglia and HMC3.

In conclusion, the effect of *A. muscaria* on neuroinflammatory cells, such as microglia, are unknown and the role of poly(I:C) signaling pathways on HMC3 cells are poorly understood. Our data show that *A. muscaria* extract AME-1 potentiated HMC3 production of pro-inflammatory cytokines in response to poly(I:C) and this effect was likely associated with trehalose, a component of the AME-1 extract, although the exact mechanism of trehalose effects on HMC3 is yet to be determined. This data suggests that the AME-1 extract may prime microglial cells toward a protective innate immune response facilitated by TLR3.

## Data Availability

The original contributions presented in the study are included in the article/supplementary materials, further inquiries can be directed to the corresponding author.
